# Closing the gap in Australian Aboriginal infant immunisation rates -- the development and review of a pre-call strategy

**DOI:** 10.1186/s12889-016-3086-x

**Published:** 2016-06-16

**Authors:** Patrick M. Cashman, Natalie A. Allan, Katrina K. Clark, Michelle T. Butler, Peter D. Massey, David N. Durrheim

**Affiliations:** Hunter New England Population Health, Newcastle, New South Wales 2287 Australia; College of Medicine & Dentistry, James Cook University, Townsville, Queensland Australia; Hunter Medical Research Institute, 1 Kookaburra Circuit, New Lambton Heights, New South Wales 2305 Australia

## Abstract

**Background:**

Improving timely immunisation is key to closing the inequitable gap in immunisation rates between Aboriginal children and non-Indigenous children. Aboriginal Immunisation Officers were employed in Hunter New England Local Health District (HNELHD), New South Wales (NSW), Australia, to telephone the families of all Aboriginal infants prior to the due date for their first scheduled vaccination.

**Methods:**

Aboriginal Immunisation Officers contacted the families of Aboriginal children born in the Hunter New England Local Health District (HNELHD) by telephone before their due immunisation date (pre-call) to provide the rationale for timely immunisation, and to facilitate contact with culturally safe local immunisation services if this was required. The impact of this strategy on immunisation coverage rates is reviewed.

**Results:**

For the period March 2010 to September 2014 there was a significant increase in immunisation coverage rate for Aboriginal children at 12 months of age in HNELHD (*p* < 0.0001). The coverage in the rest of NSW Aboriginal children also increased but not significantly (*p* = 0.218). Over the full study period there was a significant decrease in the immunisation coverage gap between Aboriginal children and non-Indigenous children in HNELHD (*p* < 0.0001) and the rest of NSW (*p* = 0.004). The immunisation coverage gap between Aboriginal and non-Indigenous infants decreased at a significantly faster rate in HNELHD than the rest of NSW (*p* = 0.0001). By the end of the study period in 2014, immunisation coverage in HNELHD Aboriginal infants had surpassed that of non-Indigenous infants by 0.8 %.

**Conclusions:**

The employment of Aboriginal immunisation officers may be associated with closing of the gap between Aboriginal and non-Indigenous infants’ immunisation coverage in HNELHD and NSW. The pre-call telephone strategy provided accelerated benefit in closing this gap in HNELHD.

## Background

The socio-economic disadvantage of Aboriginal and Torres Strait Islander peoples in Australia is evidenced by the gap in measurable social, economic and health indicators between Aboriginal and Torres Strait Islander peoples and the rest of the Australian population [1, 2]. In 2008 the Council of Australian Governments adopted the “Close the Gap” [2] initiative. The Hunter New England Local Health District (HNELHD) in northern New South Wales (NSW), Australia has implemented a Close the Gap approach to decrease the gap between Aboriginal and non-Aboriginal children’s immunisation rates.

Higher rates of vaccine preventable diseases such as *Haemophilus influenzae* b disease, rotavirus disease, mumps, meningococcal disease, invasive pneumococcal disease and hepatitis B infection are seen in Aboriginal and Torres Strait Islander infants [3, 4]. Increasing immunisation coverage and the timeliness of immunisations is key to preventing these diseases and their attendant morbidity [4]. The National Aboriginal and Torres Strait Islander Health Plan 2013–2023 has recognised immunisation as a priority and placed particular focus on increasing awareness and immunisation uptake amongst Aboriginal and Torres Strait Islander people [5].

There are eight Local Health Districts in NSW, and in the HNELHD there are 46,954 Aboriginal people, which comprises 22.5 % of the Aboriginal population in NSW. In HNELHD, 25.0 % of the Aboriginal population is under 15 years, compared to 12.2 % of the non-Aboriginal population [6]. Prior to the immunisation initiative in HNELHD, described in this report, the fully immunised rate at 12–15 months of age was only 81.9 % for Aboriginal children compared to 93.6 % in non-Indigenous children.

The strategy to address the coverage gap described here is based on the recruitment of Aboriginal Immunisation Officers to contact all Aboriginal families prior to the due date of their infants’ first immunisation. The Aboriginal Immunisation Officers provide the rationale for timely immunisation and facilitate contact with culturally appropriate local services. The impact of this strategy on immunisation coverage rates is reviewed.

## Methods

We reviewed the impact of introducing Aboriginal Health Workers to implement an immunisation pre-call service.

**Fig. 1:**
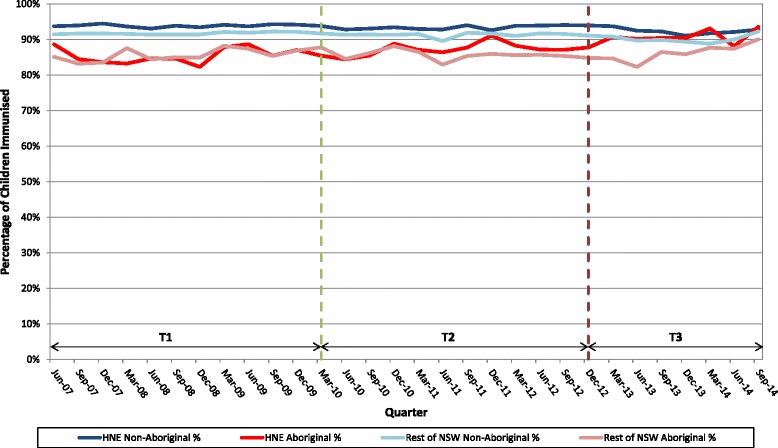
Percentage of 12–15 month old children fully immunised by Indigenous status, HNELHD and rest of NSW, by quarter, June 2007–September 2014

The full study period, June 2007 to September 2014, (Fig. 1) was divided into three time periods mirroring three distinct phases of the initiative.

Time period 1 (June 2007–February 2010): this was the period when separate Aboriginal data became available from ACIR for health planning purposes. Although there were some efforts by public health authorities and immunisation service providers to improve Aboriginal immunisation rates, there were no specific strategies employing Aboriginal people in a public health role, during this time.

Time period 2 (March 2010–November 2012): a part-time Aboriginal Health Worker began piloting the pre-call program in the HNELHD to ensure a culturally sensitive approach to addressing the immunisation coverage gap, but there were no Aboriginal immunisation officers working in other districts in the rest of NSW.

Time period 3 (December 2012–September 2014): the HNELHD program was expanded to two full-time Aboriginal Health Workers and Aboriginal Health Workers were appointed across all Local Health Districts (LHD’s) in NSW, but with only HNELHD utilizing a pre-call strategy, with over 1,000 telephone calls to the families of Aboriginal children and approximately 5,000 text messages sent out annually.

All children born in NSW during the study period who were identified as Aboriginal or Torres Strait Islander were included in the study. In HNELHD the employment of an Aboriginal Immunisation Officer revealed that the system in maternity hospital units for the identification of Aboriginal and Torres Strait Islander infants was inaccurate. The Aboriginal Immunisation Officer observed that reporting of Aboriginal identity in the maternity hospitals birth records did not reflect her own knowledge of Aboriginal community members and their newborn infants. A systematic solution was found with all new mothers specifically asked to identify the Aboriginal status of their newborn infants rather than relying on the electornic record of existing family members. This more correct data was entered into the infant’s electronic health service record before discharge. Demographic data and telephone contact details for the parent/s were then automatically provided weekly to the HNELHD Immunisation Unit [7].

The delivery of the intervention was commenced by telephone call to the family when the infant was 28 days of age; 2 weeks prior to the first immunisation encounter due at 6 weeks of age in Australia. The birth notification data was received under the Authority of the HNELHD Aboriginal Health and Well Being Alliance with representation from HNELHD and the Aboriginal Community Controlled Health Services. The Alliance recognised the immunisation gap and directed the HNELHD Immunisation Unit to address this inequity. During the first telephone call to the parents, verbal consent was obtained and recorded for future contact and ongoing vaccination reminders by SMS. The pre-call immunisation service and the evaluation did not require additional ethics approval, as it was considered a quality assurance evaluation by the HNE Human Ethics Research Committee.

All data were entered into a customised Microsoft Access database which allowed a subsequent text message (SMS) reminder to be sent prior to the scheduled immunisations at 4, 6, 12 and 18 months of age. The Aboriginal Immunisation Workers received training in immunisation and had a registered nurse available to assist with clinical questions. Whilst following a regular pattern the telephone calls were unstructured allowing relevant responses to family questions. The telephone call at 28 days of age (pre-call) served as a prompt to families to immunise their child, and provided an opportunity to discuss the importance of timely immunisation. In addition the pre-call included information about the availability of local, culturally appropriate immunisation service providers and transport, and provided information on how to make an appointment for immunisation. The Aboriginal Immunisation Officer also offered to send the link to a web-based immunisation application (http://www.immunisation.health.nsw.gov.au) to the family member’s mobile telephone if desired.

The data available for analysis was from the Australian Childhood Immunisation Register (ACIR). De-identified immunisation coverage rates by Aboriginal status and Local Government Area (LGA) are available from the ACIR to Public Health Authorities for service planning and evaluation purposes. The ACIR is a national register that records details of vaccinations given to children under 7 years of age who live in Australia. Immunisation details are sent to the ACIR by recognized immunisation providers [8]. Improved Indigenous identification on the ACIR means that vaccination coverage estimates for Indigenous children on ACIR are reliable [9]. It is estimated that the ACIR includes nearly 99 % of Australian children [10].

Quarterly (three monthly) data is routinely available from the ACIR on immunisation rates, by birth cohorts at 12–15 months of age, 24–27 months of age and 60–63 months of age. A child recorded as fully immunised at 12–15 months of age implies recorded receipt of three doses of Diphtheria Tetanus Pertussis, polio, hepatitis B, *Haemophilus* influenza type B and pneumococcal vaccines. If any scheduled dose is missed then the child is reported as not being fully immunised [11].

The routine quarterly ACIR coverage data for HNELHD and the rest of NSW were analysed using the chi-square test to determine differences in rates following implementation of the Aboriginal Immunisation Officer employment and pre-call strategy. The coverage data for Aboriginal and non-Indigenous children born in the HNELHD at quarterly endpoints were analysed and compared to the rates in the remainder of NSW. Trends in coverage rates for HNELHD and the rest of NSW were explored using z test for proportion, linear regression and the chi square test for trend (STATA 11.0).

**Fig. 2:**
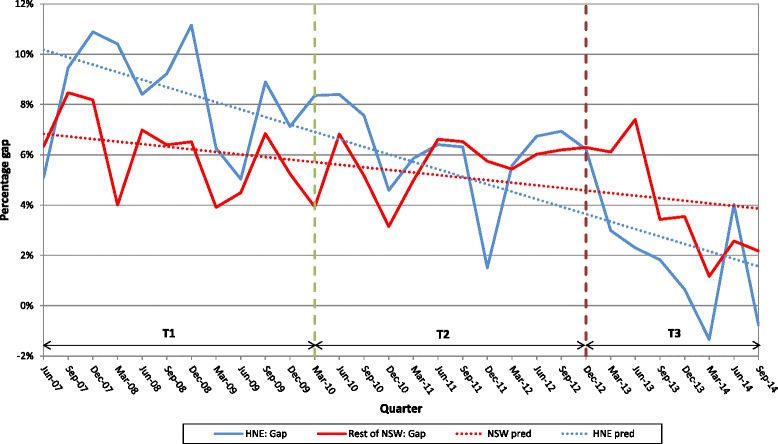
Percentage gap and predicted gap between Non-Aboriginal children fully immunised at 12–15 months of age and Aboriginal children fully immunised at 12–15 months of age, by quarter, HNELHD and rest of NSW, June 2007–March 2014

Gap analysis across the three time periods was conducted using the non-Indigenous immunisation rate as the target rate for the Aboriginal immunisation program (Fig. 2). The expected rate of Aboriginal children’s immunisation was calculated by applying the percentage of the non-Indigenous children’s immunisation rate to the population number of Aboriginal children for the three time periods. The expected number of children not fully immunised at 12–15 months of age was then calculated.

## Results

During the full study period, June 2007 to September 2014, 6,222/7,086 (87.8 %) Aboriginal children 12–15 months of age were fully immunised in HNELHD and in the rest of NSW 19,138/22,289 (85.9 %) of Aboriginal children 12–15 months of age were fully immunised.

For the full study period there was a significant decrease in the immunisation coverage gap between Aboriginal children and non-Indigenous children in HNELHD from 8.4 to 1.4 % (*p* < 0.0001). For the rest of NSW the gap decreased from 6.1 to 3.8 % (*p* = 0.004). (Table 1) Using linear regression, a significant increase in immunisation coverage for Aboriginal children in HNELHD (*p* < 0.0001) was found for time periods 2 and 3 compared to time period 1, but not for the rest of NSW (Fig. 1).

**Table 1:** Mean and range of the three-monthly percentage of fully immunised children 12–15 months of age, HNELHD and rest of NSW, over three time periods June 2007 to September 2014

Time Period		Percentage HNELHD 12–15 month olds fully immunised			Percentage rest of NSW 12–15 month olds fully immunised			*p* values for gap^a^
	Fully immunised	Non-Aboriginal	Aboriginal	Gap	Non-Aboriginal	Aboriginal	Gap	
T1 (Jun 07–Dec 09)	mean	26,232/27,944 (93.9 %)	1,913/2,238 (85.5 %)	8.4 %	206,207/224,803 (91.7 %)	6,191/7,232 (85.6 %)	6.1 %	0.437
	range^b^	93.1–94.5 %	82.3–88.7 %		91.4–92.2 %	83.2–88.2 %		
T2 (Mar 10–Dec 12)	mean	28,803/30,824 (93.4 %)	2,408/2,758 (87.2 %)	6.2 %	229,221/251,010 (91.3 %)	7,732/9,020 (85.7 %)	5.6 %	0.060
	range^b^	92.6–94 %	84.4–91.1 %		89.6–91.9 %	83.0–88.2 %		
T3 (Mar 13–Sep 14)	mean	16,674/18,063 (92.3 %)	1,901/2,090 (90.9 %)	1.4 %	132,772/147,366 (90.1 %)	85,215/6,037 (6.3 %)	3.8 %	0.752
	range^b^	91.1–93.7 %	88.1–93.6 %		88.8–92.3 %	82.3–90.1 %		
*p*-value				0.0001			0.004	

^a^calculated via Wald test comparing gradients of each line within time periods

^b^range reflects quarterly cohort coverage provided by the Australian Childhood Immunisation Register

The gaps between Aboriginal and non-Indigenous average rates across the three time periods were 8.4, 6.2 and 1.4 % for HNELHD, and 6.1, 5.6 and 3.8 % for the rest of NSW. There was a 25.8 % reduction in the gap from time period 1 to time period 2 for HNELHD and a 9.0 % reduction for the rest of NSW. From time period 2 to time period 3 there was a reduction of 77.7 % for HNELHD and 32.3 % for the rest of NSW (Table 1). The coverage gap decreased at a significantly faster rate in HNELHD than the rest of NSW (*p* = 0.0001) (Fig. 2).

During time period 1, the HNELHD Aboriginal immunisation rates for infants (mean = 85.5 %; range: (82.3–88.7 %)) were similar to the rates for the rest of NSW (mean = 85.6 %; range: (83.2–88.2 %); F(1,10); *p* = 0.1419). There was no significant difference in the immunisation coverage gap between HNELHD and the rest of NSW for Aboriginal children fully immunised at 12 to 15 months of age for time period 1 (F(1,10); *p* = 0.4372) (Fig. 2).

During time period 2 the HNELHD immunisation rates for Aboriginal infants (mean = 87.2 %; range: (84.4–91.1 %)) increased significantly (F(1,10), *p* = 0.0063) when compared to the immunisation rates for Aboriginal children in the rest of NSW (mean = 85.7 %; range: (83.0–88.2 %)) during this period (Fig. 2).

During time period 3 both the HNELHD Aboriginal immunisation rates (mean = 90.9 %; range: (88.1–93.6 %)) and those in the rest of NSW (mean = 86.3 %; range: (82.3–90.1 %)) increased and no significant difference in the slopes was found ((F1,6), *p* = 0.1716).

Using the immunisation rate of non-Aboriginal children, the number of Aboriginal children for HNELHD who were not fully immunised at 12–15 months of age in the three time periods, were 188, 170 and 28 children, a statistically significant decreasing trend (*p* = 0.0184). In time period 3 HNE also had a significantly greater proportion of the expected children immunised compared to the rest of NSW (*p* < 0.0001) (Table 2). By the end of the study period in 2014 the immunisation coverage of Aboriginal infants in HNELHD had surpassed that of non-Indigenous infants by 0.8 % (93.6 and 92.8 %).

**Table 2:** Number of Aboriginal children not fully immunised but expected to be immunised^a^ (Percent), by location, June 2007 to September 2014

Time Period	Area	Total Aboriginal children 12–15 mths of age cohorts combined	Immunisation rate non-Aboriginal children	Number of Aboriginal children expected to be fully immunised^a^	Number of Aboriginal children expected to be immunised^a^ but not fully immunised. (Percent)
June 2007–Feb. 2010	HNE	2,238	93.9 %	2,101	188 (8.95 %)
	Rest NSW	7,232	91.7 %	6,634	443 (6.68 %)
March 2010–Nov. 2012	HNE	2,758	93.4 %	2,577	169 (6.56 %)
	Rest NSW	9,020	91.3 %	8,237	505 (6.13 %)
Dec. 2012–Sept. 2014	HNE	2,090	92.3 %	1,929	28 (1.45 %)
	Rest NSW	6,037	90.1 %	5,439	225 (4.14 %)

^a^at 12–15 months of age based on the non-Aboriginal immunisation rate

## Discussion

There has been a significant closing of the immunisation gap over the 4 year life of the program since March 2010. The strategy of employing Aboriginal Health Workers in NSW appears to have been highly successful in closing the gap between Aboriginal infants and the rest of the community.

Analysis of the average gap indicates a greater change in HNELHD when compared to the rest of NSW for Aboriginal immunisation at 12–15 months of age. The systematic pre-call strategy may have contributed to the greater improvement in HNELHD and needs further exploration over time. A fully functioning pre-call strategy was the key approach utilised by HNELHD. The full implementation between December 2012 and March 2014 saw the gap in immunisation rates decrease by more than 6 % in the HNELHD. The Aboriginal Immunisation Officers in HNELHD report that the acceptability to Aboriginal families of telephone calls prior to immunisation was further enhanced by changing the health department telephone settings of ‘private number’, to have the phone number displayed to the parent.

There is good access to immunisation services for Aboriginal people across HNELHD through Aboriginal Medical Services, Aboriginal Community Controlled Health Organisations, General Practice and free public clinics. Moreover, in areas with more limited access, a very successful home visiting service exists for providing obstetric and child health care for Aboriginal families. However this access predated the current Aboriginal immunisation employment and pre-call program indicating that further strategies were required to close the immunisation gap.

Vaccines are provided free of charge under the National Immunisation Program and there are financial incentives for families whose children are fully immunised. The key challenge appears to be the need to actively link Aboriginal families who have newborns to existing culturally safer services prior to infants becoming overdue for immunisation. The initial contact of the pre-call strategy being verbal (telephone call) rather than written (overdue letter) may facilitate trust, providing a safe place for discussion about immunisation and other health issues, identifying any barriers to immunisation and facilitate access to services. This approach is likely to be more empowering for the parents of the Aboriginal infants.

A pre-call program has been evaluated in New Zealand where it was similarly found to be effective when there was also linking of the family with general practice. The New Zealand program sent a letter when the infant was 4 weeks of age followed by a phone call at 5 weeks of age if no immunisation appointment had been made. The HNELHD program included a phone call when the infant was 4 weeks of age as the initial contact [12].

There are a variety of influences that probably contributed to improving childhood immunisation rates in NSW such as Commonwealth financial incentives (www.humanservices.gov.au/customer/subjects/immunising-your-children) and the successful NSW ‘Save the Date’ immunisation advertising campaign and smartphone app (http://www.immunisation.health.nsw.gov.au). Although the state and national programs and incentives will have contributed to the improved Aboriginal immunisation rates, they worked across Aboriginal and non-Indigenous populations, so they cannot account for the differences found in HNELHD. HNELHD is distinguished from other LHDs as it has a formal partnership arrangement through the Alliance which of itself may have impacted on immunisation rates given the influence of the body.

There have been other successful strategies in Australia to improve Aboriginal immunisation timeliness and rates, which demonstrate that personalised and targeted strategies can be effective. Personal contact between Aboriginal staff and parents of children has been shown to be an important aspect of targeted immunisation programs [13]. Personalising health messages also appeared to improve the timeliness of Aboriginal infant immunisation [14]. As mobile phone use is high in Aboriginal people not living in remote areas of Australia, the use of mobile phone or SMS reminders appears to be a useful strategy that deserves further analysis [15].

In December 2013 pneumococcal vaccine (Prevenar 13) was added to the definition of fully immunised at 12 months of age which resulted in the decrease in non-Indigenous immunisation rates from December 2013. The new standard of fully immunised was subsequently harder to meet. However the same definition of fully immunised applied to Aboriginal children at 12 months of age and the improvement in Aboriginal immunisation rates occurred despite the trend of falling rates.

The HNELHD pre-call program is consistent with the principles of equity, community engagement, partnership and accountability as is enshrined in the National Aboriginal and Torres Strait Islander health plan 2013–2023 [5]. Our assessment is that the 28-day telephone call helps to empower people to make an appointment to have the infant immunised. Where necessary the HNELHD program also assists Aboriginal families to negotiate the health system. Further qualitative work evaluating the benefit of the pre-call program for the parents of the infants being immunised is certainly indicated.

## Conclusions

The gap in immunisation coverage rates between Aboriginal and non-Indigenous children persisted in the HNELHD until the employment of Aboriginal Immunisation Officers provided a change of approach and a culturally safer collaboration with Aboriginal families. The Aboriginal Immunisation Officers implemented the pre-call strategy enabling them to link families to culturally safe immunisation services prior to vaccination schedule points. This resulted in an improvement in immunisation coverage rates and a closing of the gap for Aboriginal infant immunisation.

## Abbreviations

ACIR: Australian Childhood Immunisation Register
HNELHD: Hunter New England Local Health District
IMR: infant mortality rate
LGA: Local Government Area
NSW: New South Wales
SMS: short message service
